# Progress in Diseases of the Blood

**Published:** 1902-11-08

**Authors:** 


### Progress in Diseases of the Blood.

(*Concluded from page* 82.)

**Pernicious Anæmia.**—W. Hunter[9] has examined microscopically sections from seven cases showing the presence of (1) a septic infection of the mouth, stomach and intestines, (2) a specific infection of the tongue marked by a form of glossitis, and he records the symptoms from 18 others. He insists that the onset of the disease is not preceded by any external cause sufficient to explain the anæmia, that with severe blood changes we find four groups of symptoms —anæmia, hæmolysis with deposits in the liver and spleen, gastro-intestinal sepsis, and a toxic state. The hæmolysis does not occur in other anæmias, and is not due to ordinary sepsis. The peculiar form of glossitis was found in every one of the 25 cases, the mucosa being so thinned that the lymphatics were exposed, and from them pure cultures of streptococcus longus were obtained. A. Goodall,[10] in a discussion of nine cases, notes that dental caries is not always present, and that the teeth of four of his patients were good. Hæmorrhages, urobilinuria, and respiratory troubles point to a grave prognosis. The average number of red cells in 11 cases when first seen was 1,049,000, and if a further reduction during the next fortnight took place there was soon a fatal ending; if they rose, a remission or chronic state followed. The red cells take up basic stains more readily than in health, and with a double stain will be purple instead of red. Megaloblasts, too, are often more numerous than normoblasts, a state which is hardly ever seen in other diseases. Lymphocytes are increased, and number about 50 per cent. of the white cells, but when febrile conditions occur the polynuclear ones increase for a time. As regards the diagnosis from cancer he remarks that in pernicious anæmia the reduction of red cells is greater, and that of hæmoglobin is less, the size of the red cells is greater, and polychromatophilia is marked and megalocytes are more numerous than normoblasts, there is lymphocytosis and no leucocytosis except when complications exist. However, during remissions the diagnosis may be difficult. Transfusion has done good in cases which are already mending. Arsenic, marrow, antistreptococcal injections and intestinal antiseptics have all been recommended.

**Leucocytosis.**—J. T. Hewetson[11] analyses 23 cases of suppurative appendicitis, and shows that 80 per cent. have a leucocytosis above 15,000, and 96 per cent. have 12,000 and upwards. In purely catarrhal cases without pus the average count did not exceed 8,600. Blood-counts such as these are of great value, since the temperature and pulse are at times most deceptive when the abscess is well walled off. Still, in rare instances, leucocytosis may be absent, and in profound and fatal toxæmia this is not uncommon. Some degree of leucocytosis is produced by a journey to hospital, by copious vomiting or diarrhœa, or by serous peritonitis, or possibly by a greatly distended and stenosed appendix. Chloroform seems to give a greater post-operative leucocytosis than ether. Tubercular disease of the kidney may be diagnosed from calculus, or empyæma from serous effusion by the presence of leucocytosis, and it may be noticed that after the opening of an empyæma the leucocytes fall very slowly. These results were based upon 1,500 blood-counts, and the writer speaks strongly of the immense value of the method in the diagnosis of obscure cases. C. J. N. Longridge[12] gives the results of examinations in 20 cases of various kinds, and points out that the counts are not infallible indications of the presence of pus, but that from them we can learn whether the progress is advancing or not. Again, if the polynuclear cells are specially increased, and number, say, 80 to 90 per cent., the probability of pus is much increased. His figures show the general reliability of blood-counts as laid down by Cabot and others. Da Costa[13] analysed 118 cases of appendicitis, and found that on the average the hæmoglobin was diminished by 30 per cent., and the red cells by half a million; some leucocytosis occurs in 35 per cent. of simple and 90 per cent. purulent cases. When 20,000 cells and upwards are found there is almost certainly pus, gangrene, or general peritonitis.

**Eosinophilia.**—C. G. Seligman and L. S. Budgeon[14] mention an extremely high count in a case of hydatid disease. The white cells numbered 17,000 and the red 6,290,000, and out of the former 57 per cent. were eosinophile. After operation and recovery, they dropped to 1 per cent. According to Bücklers all kinds of helminthides may bring about an excess of eosinophiles, and he records a case due to the ankylostoma where they reached 72 per cent. Another due to trichinosis numbered 68 per cent. Asthma comes nearest to these with 53·6 and 38 per cent. Pemphigus, splenectomy, some diseases of the liver and of the female genitals, and filaria are also credited with causing an excess, and Webb lays stress on the presence of some increase of eosinophiles in the diagnosis of leukæmia.

**Hæmophilia.**—C. E. Wallis[15] gives two cases where tooth extraction was needed, and though both patients were in the habit of bleeding excessively from slight wounds, a course of calcium chloride enabled the teeth to be removed without difficulty. On one occasion the patient had omitted the calcium for a week beforehand, and severe bleeding followed. The doses given were 10 to 15 grains three times a day.

[9] Lancet, May 24. [10] Scot. Med. Jour., April. [11] Birm. Med. Rev., March, April. [12] Lancet, July 12. [13] Med. Chron., Jan. [14] Lancet, June 21. [15] Brit. Med. Jour., May 10.

